# Type II metacaspase mediates light-dependent programmed cell death in *Chlamydomonas reinhardtii*

**DOI:** 10.1093/plphys/kiad618

**Published:** 2023-11-16

**Authors:** Lou Lambert, Félix de Carpentier, Phuc André, Christophe H Marchand, Antoine Danon

**Affiliations:** Institut de Biologie Paris Seine, UMR 7238, CNRS, Sorbonne Université, Paris 75005, France; Institut de Biologie Paris Seine, UMR 7238, CNRS, Sorbonne Université, Paris 75005, France; Doctoral School of Plant Sciences, Université Paris-Saclay, Saint-Aubin 91190, France; Institut de Biologie Paris Seine, UMR 7238, CNRS, Sorbonne Université, Paris 75005, France; Institut de Biologie Paris Seine, UMR 7238, CNRS, Sorbonne Université, Paris 75005, France; Institut de Biologie Physico-Chimique, Centre National de la Recherche Scientifique (CNRS), Paris F-75005, France; Institut de Biologie Paris Seine, UMR 7238, CNRS, Sorbonne Université, Paris 75005, France

## Abstract

Among the crucial processes that preside over the destiny of cells from any type of organism are those involving their self-destruction. This process is well characterized and conceptually logical to understand in multicellular organisms; however, the levels of knowledge and comprehension of its existence are still quite enigmatic in unicellular organisms. We use Chlamydomonas (*Chlamydomonas reinhardtii*) to lay the foundation for understanding the mechanisms of programmed cell death (PCD) in a unicellular photosynthetic organism. In this paper, we show that while PCD induces the death of a proportion of cells, it allows the survival of the remaining population. A quantitative proteomic analysis aiming at unveiling the proteome of PCD in Chlamydomonas allowed us to identify key proteins that led to the discovery of essential mechanisms. We show that in Chlamydomonas, PCD relies on the light dependence of a photosynthetic organism to generate reactive oxygen species and induce cell death. Finally, we obtained and characterized mutants for the 2 metacaspase genes in Chlamydomonas and showed that a type II metacaspase is essential for PCD execution.

## Teaser

*S*-nitrosoglutathione induces programmed cell death in Chlamydomonas; this process involves nitric oxide and singlet oxygen production and is dependent on light and type II metacaspase.

## Introduction

Programmed cell death (PCD) is a fundamental process in which a cell self-destructs to ensure the proper functioning of an organism, both at times during normal development and under conditions of biotic or abiotic stress. PCD is well characterized in multicellular organisms, but much less so in unicellular organisms. However, in the unicellular alga Chlamydomonas (*Chlamydomonas reinhardtii*), PCD has been shown to exist, based on criteria specifically defined in animal PCD (de Carpentier et al. 2019). It has also been shown that the self-destruction of Chlamydomonas cells, through the release of molecules in the culture medium, allowed the other cells of the population to grow better and to resist stress better (Moharikar et al. 2006; Durand et al. 2011). Thus, for Chlamydomonas, the rationale for PCD must be analyzed at the scale of population survival, as it is through the survival of the organism for multicellular beings.

The execution of apoptosis, the best-known example of PCD, is a fundamental and irreversible set of steps that are highly controlled. In animals, members of a particular family of the C14 type proteases, the caspases, act in cascade to target a variety of proteins whose degradation will allow the cell to be dismantled (Minina et al. 2020). Caspases exist almost exclusively in animals, whereas in plants and unicellular organisms, the family of C14 proteases is represented in a large majority by metacaspases (that are absent in animals), which are considered the ancestors of the caspases (Minina et al. 2017). Metacaspases are organized in several types according to the domains they possess; in plants as in most other organisms, there are type I and type II metacaspases (Minina et al. 2017), type III being represented only in phytoplankton (Choi and Berges 2013; Klemenčič and Funk 2018). It is during a study unraveling the pathogen response in Arabidopsis (*Arabidopsis thaliana*) that discovered that a type I metacaspase controls PCD (Coll et al. 2010). Since then, other papers have shown that different types of plant metacaspase (Huh 2022) and a yeast type I metacaspase (Hill et al. 2014) are also involved in this process. The role and contribution of the different metacaspases is quite complex to study in *A. thaliana*, since there are 9 metacaspases, 3 of type I and 6 of type II (Huh 2022). In Chlamydomonas, the situation is much simpler since there is only 1 type I metacaspase (MCA-I, Cre12.g517451) and 1 type II metacaspase (MCA-II, Cre03.g184700) (de Carpentier et al. 2019). MCA-I was shown to have metacaspase activity (van Midden et al. 2021), and it was also possible to purify MCA-II (Sabljić et al. 2022). But until now, the potential function of these proteins has never been described in Chlamydomonas.

Nitric oxide (NO) appears to have an important role in controlling the stress response in photosynthetic organisms mainly through its ability to induce protein S-nitrosylation. Moreover, NO has been shown to be necessary for the induction of PCD induced by high light (Chang et al. 2013) or mastoparan (MP) (Yordanova et al. 2010) in Chlamydomonas. To study PCD in Chlamydomonas, we set up a nitrosative stress-based induction system using S-nitrosoglutathione (GSNO). GSNO is considered as the main NO reservoir of the cell, and it has been shown to be a potent inducer of protein S-nitrosylation in Chlamydomonas (Morisse et al. 2014). We showed that GSNO induces PCD in Chlamydomonas. We then used a quantitative proteomic approach to identify proteins potentially involved in GSNO-induced PCD. The result of this analysis and other experiences that we describe in this article allowed us to uncover fundamental features of PCD execution in Chlamydomonas. First, this process is light-dependent and uses the chlorophyll synthesis pathway to produce ROS, and second, it requires MCA-II, a type II metacaspase.

## Results

### GSNO induces PCD in Chlamydomonas

**Figure 1.**
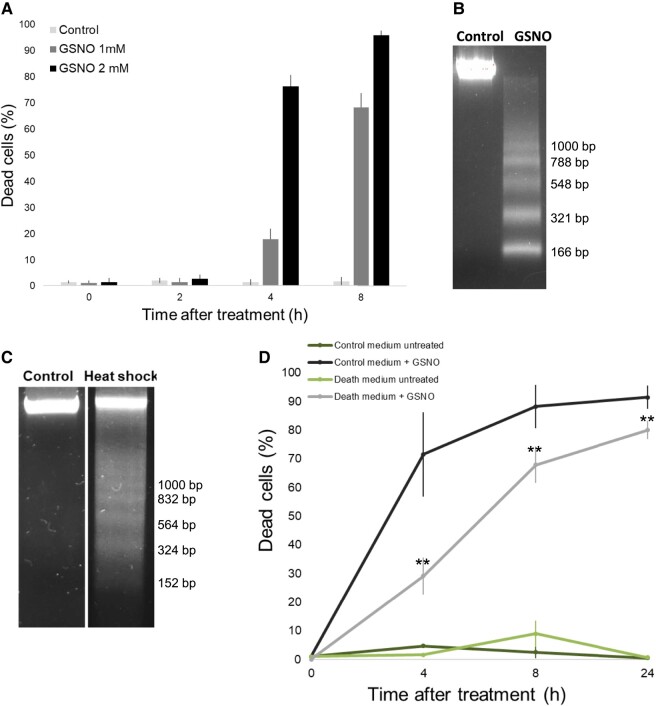
Characterization of GSNO-induced death. **A)** Measurement of the percentage of dead cells at different times after treatment with 2 concentrations of GSNO, using Evans blue. Values represent the average of 6 biological samples; error bars indicate ± Sem. **B, C)** Analysis of DNA fragmentation (10 *µ*g) by electrophoresis of cells treated with GSNO (1 mM) or heat shock (50 °C, 3 min), in comparison with the control sample. **D)** Analysis of the impact of the control medium compared with the medium where cells were killed by PCD (heat shock, 50 °C, 3 min), on the percentage of dead cells at different times after GSNO induction (1 mM). Values represent the average of 6 biological samples; error bars indicate ± Sem and for *t* test: **P ≤ 0.01.

To assess the degree of GSNO toxicity in Chlamydomonas cultures, we treated them with 1 or 2 mM and calculated the fraction of dead cells at different times after treatment. For both concentrations, we observed the induction of death in comparison with the untreated cells. However, the 1 mM concentration was more suitable to study this phenomenon since it resulted in a lower number of dead cells and more progressive cell death over time (Fig. 1A). To assess the type of cell death induced by GSNO, we analyzed the DNA degradation profile 8 h after treatment, which corresponded to about 70% dead cells. Figure 1B clearly shows that GSNO induces internucleosomal DNA degradation, which is illustrated by a DNA ladder with an average rung size of 181.6 bp, corresponding to the size of a nucleosome (Lodha and Schroda 2005). The internucleosomal degradation of DNA is specific to PCD, in contrast to necrosis which induced a random degradation of DNA that results in the appearance of a smear (Danon et al. 2000). To further analyze the role of PCD in Chlamydomonas, a unicellular organism, we studied the impact of the culture medium where cells had died. Instead of using a chemical-induced stress and thus residual molecules that could still induce PCD in the culture medium, we did not induce death with GSNO, but with a heat shock at 50 °C for 3 min, a treatment also capable of inducing PCD, as revealed by the internucleosomal degradation of DNA with an average rung size of 182 bp (Fig. 1C). Twenty-four hours after PCD induction by heat shock, the culture medium was collected after centrifugation and filtration to be sure to remove all remaining cells. Cells from a fresh culture were then pelleted by centrifugation and resuspended, either in their own culture medium or in the PCD culture medium, in which heat shock–stressed cells had previously died. The 2 cultures were then treated with GSNO to induce PCD. We could observe that both media did not induce death by themselves but, more interestingly, the PCD medium was able to strongly slow down GSNO-induced PCD, particularly at 4 h when around half the cells had died compared with control (Fig. 1D).

### Quantitative proteomic analysis

**Figure 2.**
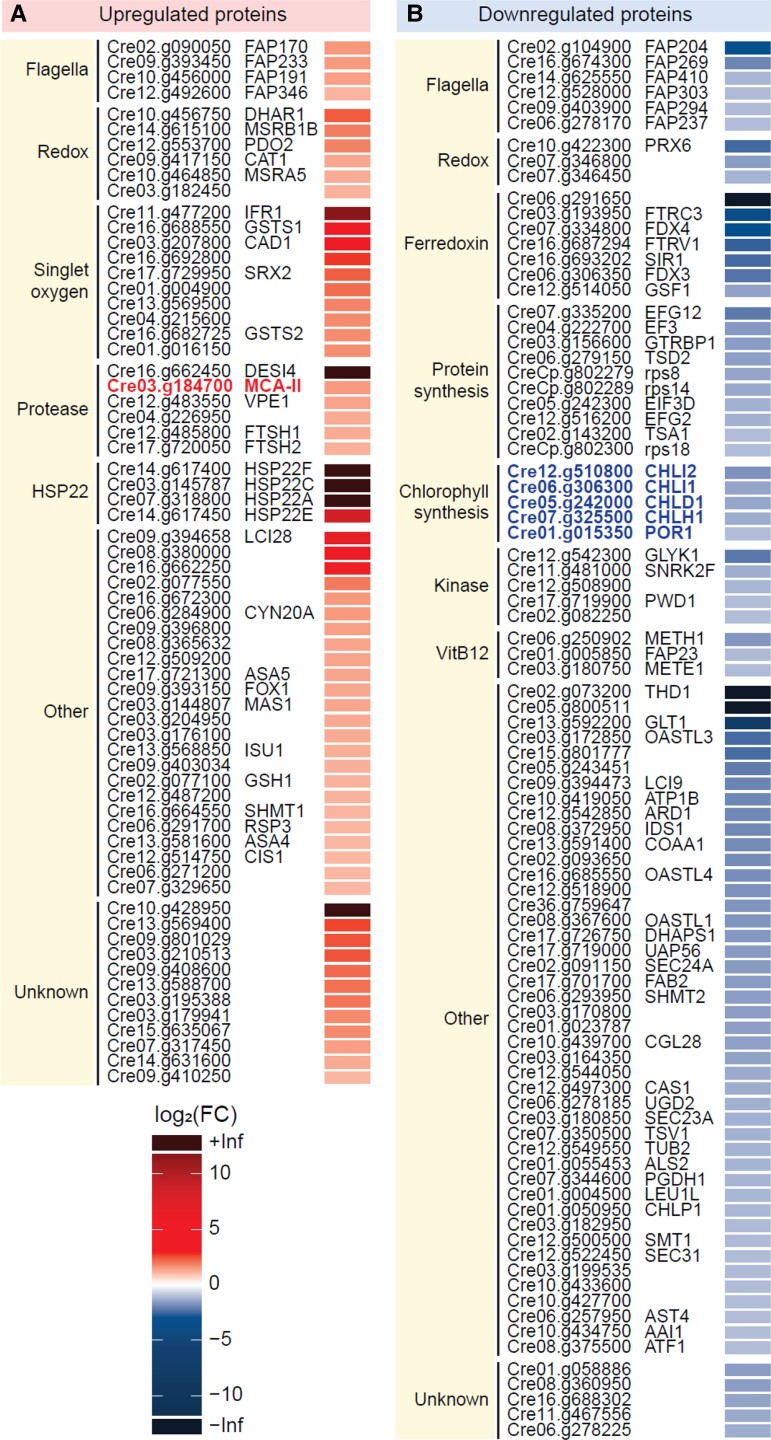
Quantitative proteomics of GSNO-induced PCD. Heat map representing proteins **A)** upregulated or **B)** downregulated by at least a factor 2 during PCD; the gene number and the name of the proteins (when available) are indicated. The families to which these proteins belong, as indicated in Phytozome (https://phytozome.jgi.doe.gov) or the literature, are also indicated in the yellow box.

**Figure 3.**
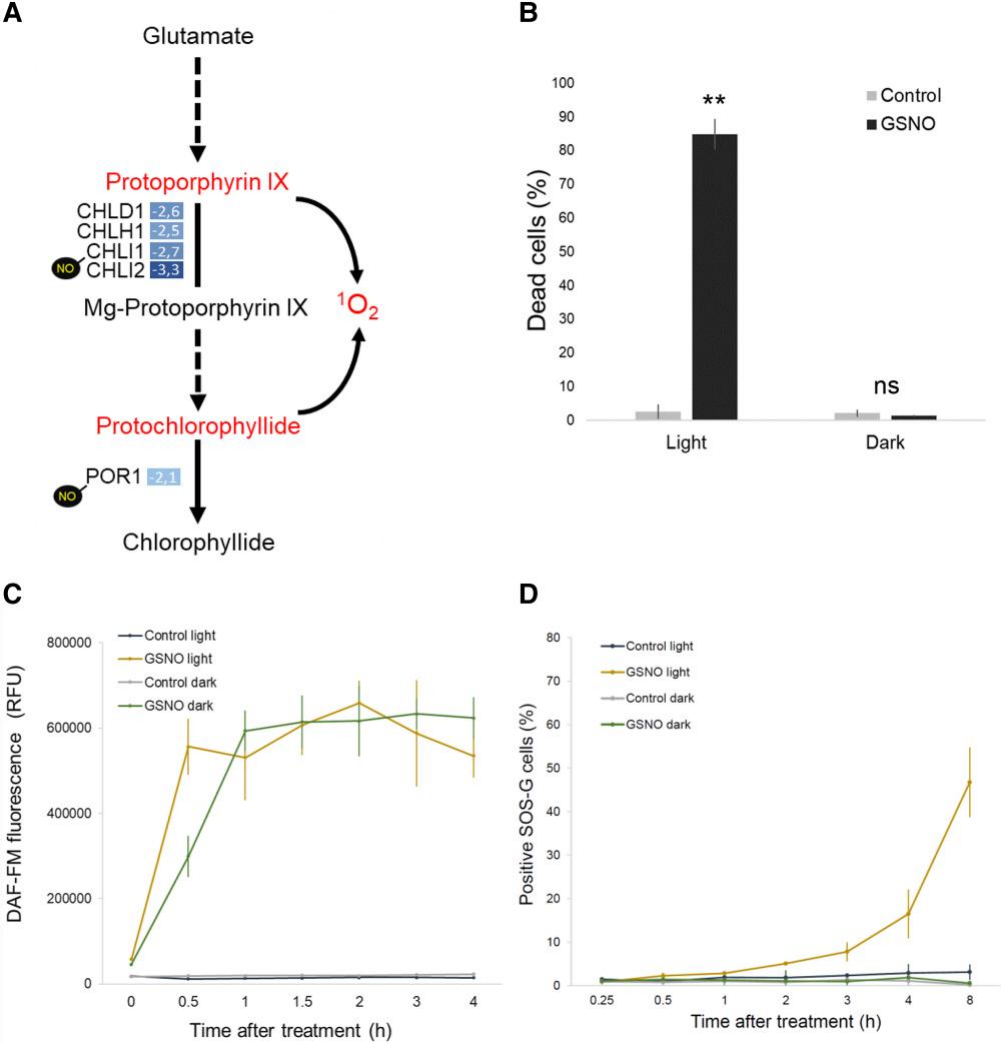
Impact of light on the death and production of NO and singlet oxygen. **A)** Model showing deregulated and possibly nitrosylated (NO) proteins of the chlorophyll synthesis pathway and possible consequences on singlet oxygen production. **B)** Impact of light on the percentage of dead cells induced by GSNO (1 mM) after 8 h. Values represent the average of 4 biological samples; error bars indicate ± Sem and for *t* test: **P ≤ 0.01. **C)** Kinetics of NO production in control or in GSNO (1 mM) treated cells under light or dark conditions. Fluorescence of DAF-FM was detected using excitation and emission wavelengths of 483 and 530 nm, respectively. Values represent the average of 4 biological samples; error bars indicate ± Sem. **D)** Kinetics of the percentage of cells producing singlet oxygen (detected by fluorescence of SOS-G), in control or GSNO (1 mM)-treated cells under light or dark conditions. Values represent the average of 4 biological samples; error bars indicate ± Sem.

To identify proteins whose quantities change during GSNO-induced PCD, we used mass spectrometry to perform untargeted quantitative proteomic experiments. We compared untreated cultures of Chlamydomonas with cultures treated with GSNO (1 mM) 5 h after treatment, since we showed that in this zone of kinetics the death process had already been started and was still in progress, as it affected only a small part of the population (Fig. 1A). During this experiment, we detected a total of 3,787 proteins in our samples, from which we identified 66 proteins that were upregulated and 88 that were downregulated by at least a factor 2 during PCD (Fig. 2, Supplemental Table S1). We classified these differentially expressed proteins into different categories, according to their description on the Phytozome website (Goodstein et al. 2012) or data collected in the literature (Fig. 2). As expected, a strong proportion of proteins corresponding to genes previously identified as being regulated by nitrosative stress (Kuo and Lee 2021) were found. In this category, among the induced proteins, a family of heat shock proteins is particularly well represented, since 4 of their members are also strongly induced during PCD (Fig. 2). In general, we found a high proportion of downregulated proteins that are predicted to be or localized in the chloroplast (48.8% versus 28.5% for upregulated proteins). Among the proteins repressed during nitrosative stress and PCD are strikingly 4 of the subunits of Mg chelatase (MgCh), which plays a key role in chlorophyll synthesis (Fig. 2B). Another protein involved in chlorophyll synthesis, protochlorophyllide reductase (POR), is downregulated during PCD. Interestingly, the downregulation of MgCh and POR should lead to the accumulation of their respective substrates, protoporphyrin IX and protochlorophyllide. The accumulation of these 2 intermediates of chlorophyll synthesis is potentially deleterious, because they can be excited by light and transmit their excitation energy to oxygen to form singlet oxygen (^1^O_2_) (op den Camp et al. 2003; Tarahi Tabrizi et al. 2016) (Fig. 3A). The production of singlet oxygen could be all the more important as the geranylgeranyl reductase (CHLP), which is involved in the synthesis of carotenoids, pigments capable of scavenging it (Tamaki et al. 2021), is also downregulated by GSNO treatment. Moreover, we have identified a large number of proteins induced during PCD that have already been described as participating in the singlet oxygen response in Chlamydomonas (Chang et al. 2014; Wakao et al. 2014; Venkanna et al. 2017) (Fig. 2). The change of the redox status of the cell could also be evidenced by the decrease of the content of several ferredoxins but also of cobalamin (vitamin B12) which is known to induce a better resistance to oxidative stress (van de Lagemaat et al. 2019) and heat shock in Chlamydomonas (Xie et al. 2013) (Fig. 2). Protein synthesis seems to be slowed down during PCD since we have identified several contributing proteins that are downregulated, which is also the case for several kinases (Fig. 2B). Finally, the protease family has particularly attracted our attention, since it is found in the control or execution of PCD in most organisms where it has been studied (Salvesen et al. 2016) (Fig. 2). In particular, we observed that a type II metacaspase, which is involved in PCD in plants (Minina et al. 2017), was upregulated during PCD in Chlamydomonas.

### The role of light and singlet oxygen in GSNO-induced PCD

To test whether the results suggested by proteomics regarding the possible production of singlet oxygen were correct, we treated the cells with GSNO (1 mM) and then we placed them either in the light or in the dark and measured the percentage of dead cells after 8 h. This experiment clearly showed that cells placed in the dark, although treated with GSNO, were well protected from PCD (Fig. 3B). As GSNO is a major NO donor in the cell, we determined the presence of NO using Diaminofluorescein-FM diacetate (DAF-FM) (Chang et al. 2013), and we quantified its fluorescence at different time points after the treatment. We could see that NO can be detected as early as 15 min after GSNO treatment and that its production then increases linearly, reaching a plateau after 60 min (Fig. 3C). We also treated the cells with GSNO but then placing them in the dark and quantified NO as we did before, and we observed that the light had little or no impact on NO production (Fig. 3C). To test whether the light dependence of GSNO-induced PCD was related to singlet oxygen, we detected its presence using the singlet oxygen sensor (SOS) probe (Prasad et al. 2016). We measured the percentage of cells producing singlet oxygen over time in light and dark, and we could see that about 3 h after GSNO treatment, singlet oxygen could be detected and that its production then increases linearly (Fig. 3D). The results also clearly showed, confirming our hypothesis, that singlet oxygen production was absent in the dark (Fig. 3D).

### Characterization of metacaspase mutants

**Figure 4.**
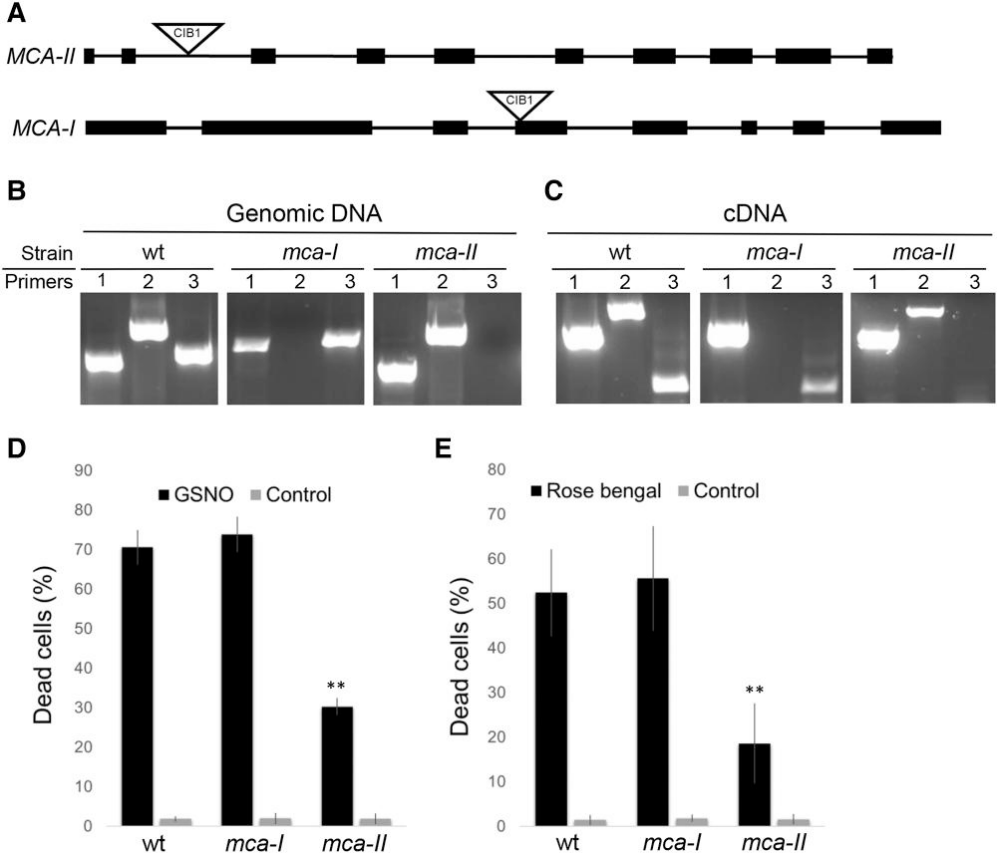
Characterization of metacaspase 1 and 2 mutants. **A)** Location of CIB1 insertions in *mca-I* (LMJ.RY0402.231745) and *mca-II* (LMJ.RY0402.135349) mutants. Lines represent introns and black rectangles represent exons. **B)** Genotyping of the wild type and *mca-I* and *mca-II* mutants using Rack1 control primers (1) or located on either side of the insertions in MCA-I (2) or MCA-II (3). **C)** Impact of CIB1 insertion on the expression level of *RACK1* (1), *MCA-I* (2), and *MCA-II* (3) genes, in wild type and *mca-I* and *mca-II* mutants, using RT-PCR. **D, E)** Measurement of the percentage of dead cells, 8 h after treatment with GSNO (1 mM) or rose bengal (10 *µ*M), in wild type and *mca-I* and *mca-II* mutants, using Evans blue. Values represent the average of 6 biological samples; error bars indicate ± Sem and for *t* test: **P ≤ 0.01.

Among the proteins that we identified as being more abundant during GSNO-induced PCD is a metacaspase (MCA-II), which belongs to a family of proteins known to be involved in PCD in plants (Minina et al. 2017). In Chlamydomonas genome, there is 1 type I MC (*MCA-I*, Cre12.g517451) gene and 1 type II MC (*MCA-II*, Cre03.g184700) gene (de Carpentier et al. 2019). In order to analyze the role that metacaspases might play in GSNO-induced PCD, we searched for mutants from the CLiP collection (Li et al. 2019), for *MCA-II*, but also for *MCA-I*. We obtained a mutant for *MCA-I* (LMJ.RY0402.231745) and a mutant for *MCA-II* (LMJ.RY0402.135349) (Fig. 4A). We then verified for both genes that there was an insertion of the CIB1 cassette at the location predicted by the CLiP library (Li et al. 2019) (Fig. 4B), which resulted in the extinction of the expression of the corresponding genes (Fig. 4C). We then tested the impact of an 8 h treatment of GSNO (1 mM), on the mortality rate of *mca-I* and *mca-II* mutants compared with the wild type. We found that in the *mca-II* mutant, more cells remain viable after the same treatment in comparison with the wild type and the *mca-I* mutant, which showed the same response (Fig. 4D). In relation to the potential role of singlet oxygen that we had identified for GSNO-induced PCD, we tested the resistance of metacaspase mutants to rose bengal which induces singlet oxygen production (Fischer et al. 2012). We found that similar to nitrosative stress, MCA-II appears to be involved in singlet oxygen–induced cell death, as the *mca-II* mutant exhibits increased resistance compared with the *mca-I* and wild-type strains (Fig. 4E).

### Overexpression of metacaspase

**Figure 5.**
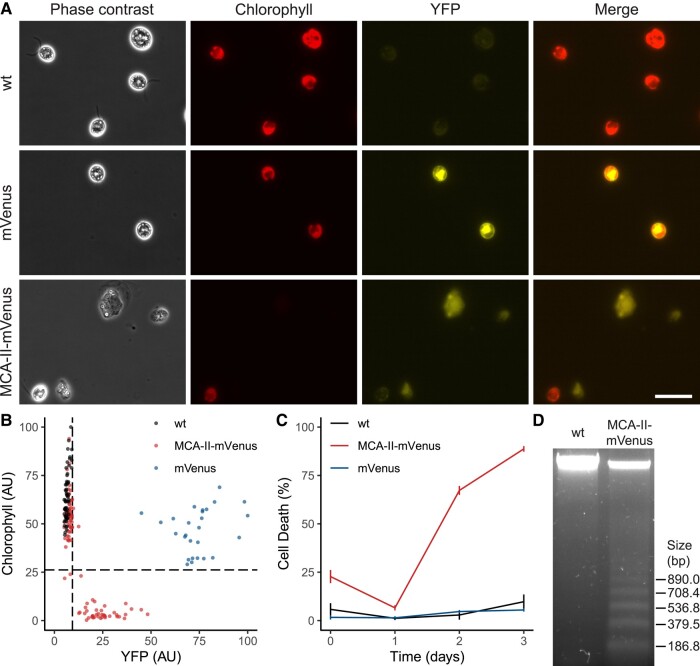
Analysis of the localization and impact of MCA-II overexpression. **A)** The wild type, the mVenus control strain, as well as the strain overexpressing MCA-II-mVenus are observed under a microscope, in phase contrast, under a wavelength of 480 nm to see the chlorophyll autofluorescence (in red) and a wavelength of 515 nm to see the mVenus fluorescence (in yellow). **B)** Representation of wild-type, mVenus, or MCA-II-mVenus cells according to chlorophyll and YFP fluorescence. Scale bar represents 50 *µ*m for all images. **C)** Percentage of dead cells in strains overexpressing wild-type, mVenus, and MCA-II-mVenus, different days after the beginning of the culture. Values represent the average of 6 biological samples; error bars indicate ± Sem. **D)** DNA analysis of strains overexpressing wild-type and MCA-II-mVenus after 3 d of culture.

The results obtained with the metacaspase mutants suggest that MCA-II may be involved in PCD execution in Chlamydomonas. To test this hypothesis, we constructed a plasmid where *MCA-II* is fused with the *mVenus* reporter gene and controlled by the strong promoter *pPsaD* (photosystem I reaction center subunit II) (Crozet et al. 2018) (Supplemental Fig. S1). We then transformed the wild-type strain with this plasmid (MCA-II-mVenus) or an mVenus positive control (mVenus). We showed that the MCA-II-mVenus strain overexpressed the *MCA-II* gene (Supplemental Fig. S1). Our microscopic observations showed us that mVenus fluorescence was present in the mVenus control and absent in the wild type (Fig. 5A). We then analyzed the MCA-II-mVenus strain and observed that MCA-II seems to be localized in the cytoplasm of Chlamydomonas (Fig. 5A), like its homolog in Arabidopsis AtMC4 (Watanabe and Lam 2011). However, MCA-II was not detected in all cells of the population, suggesting that it is not continuously expressed or stable during the cell cycle (Fig. 4B). Indeed, in the cells where we detected MCA-II, we observed that most of the time chlorophyll was no longer detectable, which is an interesting phenotype since its disappearance is considered in plants as a marker of cell death (Xi et al. 2021). We have analyzed this phenomenon in detail in Fig. 5B, where we show that unlike the mVenus strain, in the MCA-II-mVenus strain, the cells exhibiting high mVenus fluorescence have very low chlorophyll-associated fluorescence values. To determine whether the loss of chlorophyll in the MCA-II-mVenus strain is related to a cell death phenomenon, we measured the rate of dead cells at different times after the beginning of the cultures of the wild-type and MCA-II-mVenus strains. This allowed us to observe that indeed, the cells overexpressing *MCA-II* die progressively during a process that takes several days (Fig. 5C). If this process takes a long time and the cells are not dead from the beginning, it’s because time 0 of this experiment corresponds to the moment when the cells preserved on agar medium are transferred to liquid culture. We have observed that when MCA-II-mVenus cells are maintained in culture on a solid support, they can be kept for several weeks, but it's only in liquid culture that the cells start to die. This is due to the properties of the PsaD promoter (Crozet et al. 2018) we use to control MCA-II-mVenus expression, as it has indeed been shown that this promoter is much less active in cells maintained on solid medium compared with liquid cultures (Bogaert et al. 2018). This enabled us to select transformants expressing MCA-II-mVenus on agar without them dying and then to analyze the same cells in liquid medium while pPsaD was fully active. The switch from solid to liquid medium thus constitutes a PsaD promoter induction system particularly well suited to the overexpression of proteins encoded by the genes toxic to Chlamydomonas. We have verified that in our particular case, MCA-II-mVenus is indeed more strongly expressed after 3 d of liquid culture, rather than on solid medium (Supplemental Fig. S2). To identify the type of death induced by *MCA-II* overexpression, we analyzed the DNA quality of wild-type and MCA-II-mVenus strains 3 d after the start of culture. This allowed us to show an internucleosomal type of DNA degradation in MCA-II-mVenus with an average rung size of 180.1 bp, characteristic of PCD (Fig. 5D).

**Figure 6.**
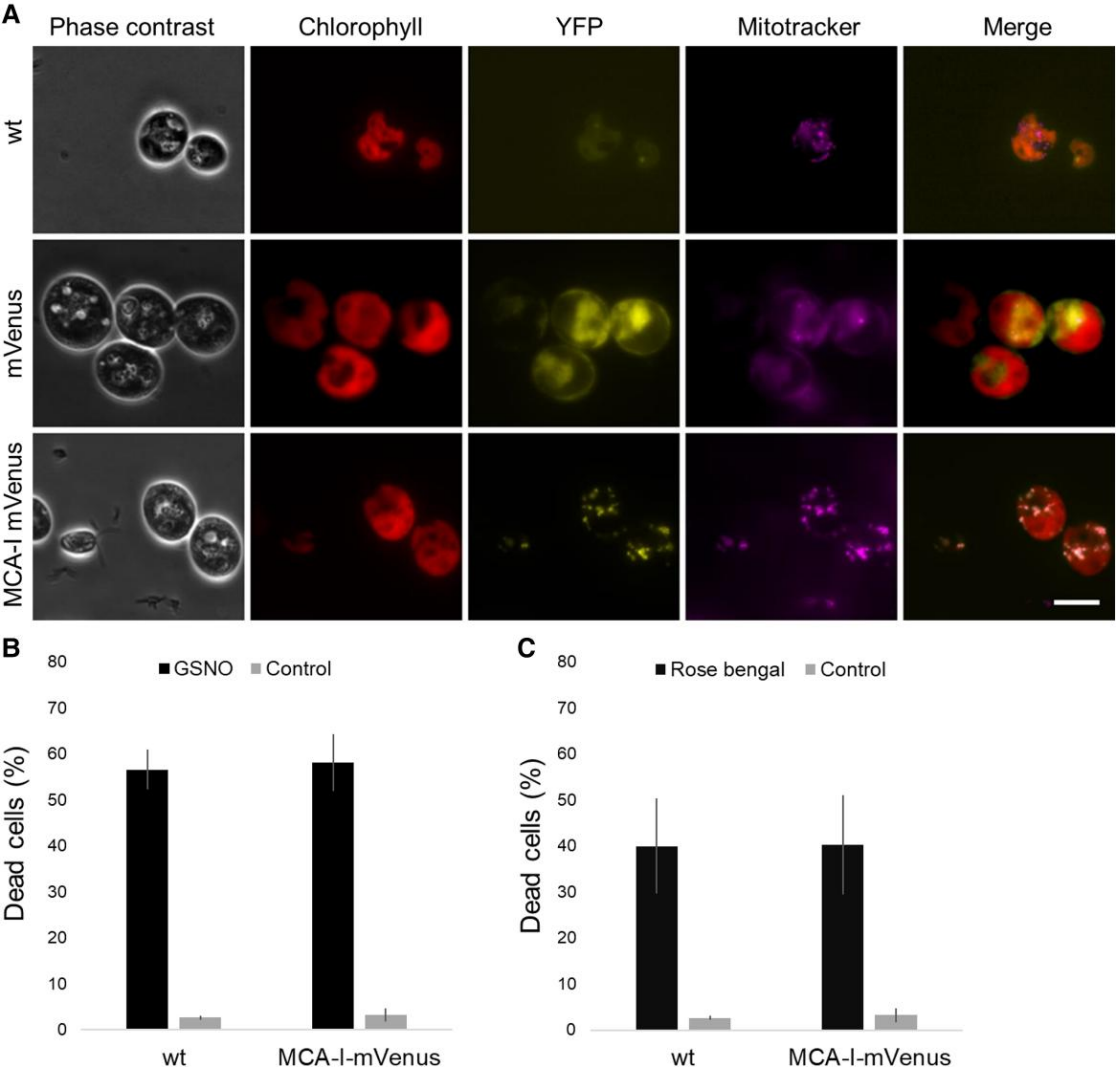
Analysis of the localization and impact of *MCA-I* overexpression. **A)** The wild type, the mVenus control strain, as well as the strain overexpressing MCA-I-mVenus are observed under a microscope, in phase contrast, under a wavelength of 480 nm to see the chlorophyll autofluorescence (in red), a wavelength of 515 nm to see the mVenus fluorescence (in yellow), and a wavelength of 579 nm to see the mitochondria (Red CMXRos, in purple). Scale bar represents 10 *µ*m for all images. **B, C)** Measurement of the percentage of dead cells, 24 h after treatment with GSNO (1 mM) or Rose bengal (10 *µ*M), in wild type and in the strain overexpressing MCA-I-mVenus, using Evans blue. Values represent the average of 8 biological samples; error bars indicate ± Sem.

In the same way as we did for *MCA-II*, we overexpressed *MCA-I* fused to mVenus (Supplemental Fig. S1). We showed that in the MCA-I-mVenus strain, *MCA-I* was really overexpressed (Supplemental Fig. S1). We localized MCA-I in Chlamydomonas cells, and we observed the fluorescence of mVenus organized as small dots in the cell, which could resemble mitochondria (Fig. 6A). To test this hypothesis, we labeled cells with the Mitotracker probe that specifically labels mitochondria. Figure 6A clearly shows that MCA-I-mVenus and Mitotracker signals overlap perfectly, indicating that MCA-I is indeed localized in the mitochondria. We then tested whether *MCA-I* overexpression had an impact on PCD, finding that it did not lead to increased death (Fig. 6B). We then showed that the strain overexpressing *MCA-I* behaved similarly to the wild-type strain in the percentage of cells killed by GSNO (Fig. 6B) or rose bengal treatment (Fig. 6C).

## Discussion

We have recently shown that in response to moderate environmental stresses, Chlamydomonas is able to form multicellular structures that can contain several thousand cells, to ensure the survival of its population (de Carpentier et al. 2022). The study of self-destruction of unicellular organisms in response to stronger stress is another fascinating way for understanding how cells can communicate to adapt to situations that are critical for their survival. In this study, we were interested in PCD induced by nitrosative stress, through the effect of GSNO that our team had already used to study protein S-nitrosylation (Morisse et al. 2014), and since it has been shown that NO is involved in PCD induced by several treatments in Chlamydomonas (Yordanova et al. 2010; Chang et al. 2013). To our knowledge, although being an essential element of regulation of the redox status of the cell, GSNO has never been studied directly in the context of PCD in any organism. We have shown that GSNO induces cell death in Chlamydomonas in a time- and dose-dependent manner and that the induced death was PCD (Fig. 1A). In addition, GSNO-induced PCD can be inhibited by the medium of other cultures killed by heat shock that also induce PCD. This illustrates the reason for being of PCD in a unicellular organism, since if the culture medium in which cells have died is able to protect new cells from PCD, it is probably because it has been enriched with protective molecules from the cells that had self-destructed. Such a mechanism of enrichment in volatile organic compounds with antioxidant activity has already been described during PCD in *Nicotiana tabacum* BY-2 cell cultures, which the authors suggest may have a protective role (Iakimova et al. 2020). It would therefore now be very exciting to study the medium of PCD-dead cells to try to identify the molecules responsible for the protective effect. The approach that we have recently developed for the study of aggregation (de Carpentier et al. 2022) could also be relevant for PCD.

**Figure 7.**
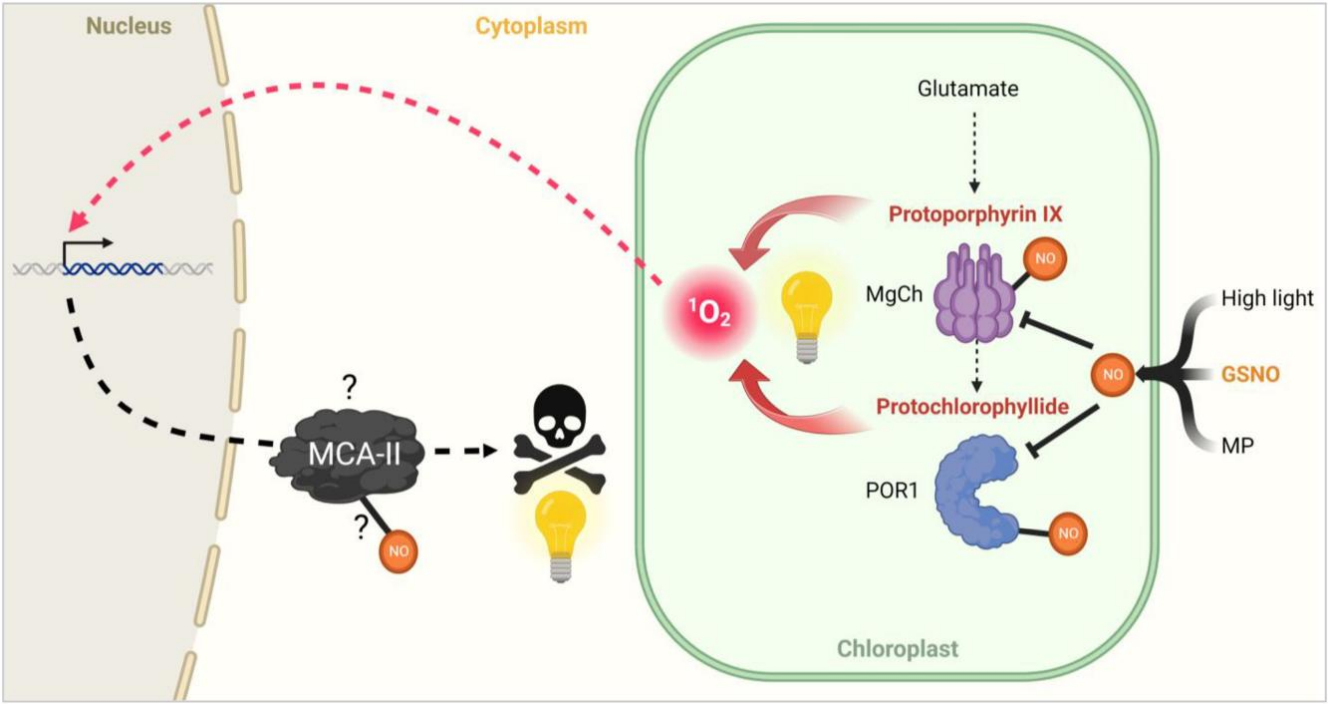
Proposed model for GSNO-induced PCD. In this model, GSNO treatment induces rapid NO production and a decrease in MgCh and POR1, potentially involving their nitrosylation. This leads to the accumulation of protoporphyrin IX and protochlorophyllide, which in the presence of light are capable of producing singlet oxygen (^1^O_2_). The ^1^O_2_ will induce the induction of specific genes leading to PCD, which may include MCA-II. This protease could also be the target of nitrosylation, as in Arabidopsis. This scheme could be valid for other types of PCD in Chlamydomonas, induced by high light or MP, which have also been shown to involve NO. The dotted lines indicate the ^1^O_2_ signaling pathway and its potential impact on MCA-II expression.

Our subsequent quantitative proteomic study, which aimed to better understand PCD in Chlamydomonas, revealed at least 2 important findings. While we logically found proteins already identified as being involved in the nitrosative stress response (Kuo and Lee 2021), intriguingly, we identified many proteins known to participate in the singlet oxygen response (Fig. 2B), suggesting that this particular ROS may be linked to GSNO-induced PCD. This hypothesis was reinforced by the fact that in our proteomic analysis, MgCh and POR 1 (POR1) were downregulated during PCD, which should lead to the accumulation of their respective substrates, protoporphyrin IX and protochlorophyllide, molecules that generate singlet oxygen in the presence of light (op den Camp et al. 2003; Tarahi Tabrizi et al. 2016) (Fig. 7). In addition, several lesion mimic mutants, which spontaneously trigger PCD in plants, have been shown to be defective in genes involved in the synthesis or degradation of chlorophyll (Bruggeman et al. 2015), linking chlorophyll synthesis or degradation intermediates to singlet oxygen production (Bhatt et al. 2021). Based on these observations, we tested the effect of light after GSNO treatment and demonstrated that, in Chlamydomonas, PCD can be light-dependent (Fig. 3B). We have further shown that while GSNO induces NO production in both light and dark (Fig. 3C), it also does induce singlet oxygen production but only in the presence of light (Fig. 3D). Therefore, NO itself is not sufficient to induce PCD, another molecule must be produced, whose effect depends on light, such as singlet oxygen. It is known that in plants the production of singlet oxygen induces PCD by well-characterized mechanisms (op den Camp et al. 2003; Wagner 2004). It is interesting to point out that in the sequence of events we have observed the production of NO which reaches a plateau after 1 h (Fig. 3C) precedes that of singlet oxygen (2 h; Fig. 3D), which itself precedes the detection of the first dead cells (4 h; Fig. 1A). We can therefore hypothesize that during GSNO-induced PCD, target proteins are deregulated, leading to the accumulation of molecules generating singlet oxygen in the presence of light, which leads to cell death. Interestingly, a transcriptomic analysis during nitrosative stress in Chlamydomonas showed that genes encoding exactly the same MgCh subunits that we identified (*CHLH*, *CHLI1*, *CHLI2*, and *CHLD*) are also downregulated (Kuo and Lee 2021). Another mode of regulation of these proteins could be S-nitrosylation, since both POR1 and CHLI1 have been shown to be targets of it (Morisse et al. 2014) (Fig. 7). S-nitrosylation is a cysteine-centered redox posttranslational modification which plays a major role in cell signaling, and GSNO is a key trans-nitrosylating agent in Chlamydomonas (Morisse et al. 2014). This mode of regulation could not be limited to GSNO-induced PCD, since PCD induced by other inducers such as MP or high light, also involve NO production (Yordanova et al. 2010; Chang et al. 2013) (Fig. 7).

The second important finding of our study concerns the role of metacaspases in the control of PCD in Chlamydomonas. We were particularly interested in this family of proteases since one of them, MCA-II, is present in higher amounts during GSNO-induced PCD, and metacaspases are considered to be the ancestors of caspases, a very well-characterized protease family specific for PCD in animals (Salvesen et al. 2016). There is no caspase in plants or unicellular organisms, but the role of metacaspases in PCD has already been demonstrated in plants (Huh 2022). We show in this paper that in Chlamydomonas, the type II metacaspase, MCA-II, is involved in PCD, since in the *mca -II* mutant death induced by GSNO or rose bengal (a singlet oxygen generator) is strongly inhibited (Fig. 4, D and E), and *MCA-II* overexpression leads to PCD. We have shown (Fig. 4C), along with other work (Zones et al. 2015), that the *MCA-II* gene is normally expressed in a quite large amounts in the cell, implying that MCA-II plays a role in cell function outside the PCD. On the other hand, when *MCA-II* is highly expressed, as may be the case in response to stress (Fig. 7), its function becomes to induce PCD. MCA-II is localized in the cytosol of Chlamydomonas cells (Fig. 5A), like its homolog in Arabidopsis, AtMC4 (Huh 2022). Our knowledge is still too limited to understand what could be the role of MCA-II in Chlamydomonas PCD, for example, there is still no link between singlet oxygen and metacaspase (Fig. 7). However, it has been shown in *A. thaliana* that the activity of the type II metacaspase AtMC9 is regulated by nitrosylation (Belenghi et al. 2007), which could make sense in relation to what we have described above (Fig. 7). We also know that in Chlamydomonas, MCA-II has been identified as a thioredoxin target (Pérez-Pérez et al. 2017). Beside their disulfide oxido-reductase activity, thioredoxins can also denitrosylate specific cysteine of proteins affected by this redox posttranslational modification (Benhar et al. 2008). To understand the function of MCA-II in PCD in Chlamydomonas, it will now be important to test the impact of nitrosylation on its activity, as well as to identify its specific targets. Little is known about the targets of metacaspases and how they function, although in Arabidopsis, for example, it has been shown that AtMC4, the homolog of MCA-II, is responsible for the activation by cleavage of plant elicitor peptide 1 (PEP1), which induces a defense response to pathogens (Shen et al. 2019). Chlamydomonas would be particularly well suited to finding metacaspase targets. A quantitative proteomic approach comparing the MCA-II mutant, its overexpressor and the wild type could open up avenues. Then, a secondary mutagenesis on the MCA-II overexpressor could also help identify the targets of this protease, by screening strains that no longer show the lethal phenotype. Interestingly, other proteases with caspase-like activities could also be involved, since a vacuolar processing enzyme (VPE) homolog whose function has been shown in Arabidopsis (Hatsugai et al. 2015) is also induced during GSNO-induced PCD in Chlamydomonas (Fig. 2). The study of VPE1 in Chlamydomonas could enable us to highlight its role in PCD and test a possible link between proteases with caspase-like activities and metacaspase.

Concerning the type I metacaspases MCA-I, our studies showed no direct involvement in GSNO- or rose bengal-induced PCD, either at the mutant or overexpressor strain level. However, it cannot be excluded that MCA-I participates in another type of PCD or in response to stress as shown in yeast (Hill et al. 2014). For example, MCA-I could be involved in the Chlamydomonas response to H_2_O_2_, since this treatment induces expression of its gene (Murik et al. 2014), whereas our study only focused on nitrosative stress (GSNO) or oxidative stress but induced by singlet oxygen (rose bengal). As the signaling pathways of singlet oxygen and H_2_O_2_ are quite different (Laloi et al. 2007), a model in response to the latter could be different from the one we found (Fig. 7) and could involve MCA-I instead of MCA-II. If the localization of type I metacaspases has already been observed in different parts of the cell in diverse organisms (Tsiatsiani et al. 2011), our results clearly show that a metacaspase is located in the mitochondria (Fig. 6A). This organelle could be particularly relevant for the stress response and PCD, since in animals in particular it is a central organelle for PCD initiation (Wolf et al. 2022), where caspase 2 is specifically localized (Lopez-Cruzan et al. 2016). Thus, our results lead the way for the study of the function of type I metacaspase. Since it has also recently been shown in Arabidopsis that a type I metacaspase has an important function in protein disaggregation during stress (Ruiz-Solaní et al. 2023), it would also be interesting to test in Chlamydomonas the mutant and overexpressor of MCA-I for this very important process for cell survival.

Our study could lay the foundation for understanding PCD in photosynthetic unicellular organisms and show that Chlamydomonas could be an excellent model organism to study the mode of action of metacaspases during PCD, since we have shown that at least MCA-II is involved (Fig. 7). Moreover, Chlamydomonas has only 1 copy of genes encoding type I and II metacaspases in its genome, in contrast to other photosynthetic model organisms, such as Arabidopsis (9 *MCAs*) (Huh 2022), rice (*Oryza sativa*) (8 *MCAs*) (Huang et al. 2015), or maize (*Zea mays*) (11 *MCAs*) (Luan et al. 2021), for example. In addition, in Chlamydomonas, metacaspases can be purified and their activity measured in vitro (van Midden et al. 2021; Sabljić et al. 2022), which will greatly facilitate the study of their function and the search for their targets, for which very little is yet known. Finally, the ability to develop genetic approaches easily and rapidly in Chlamydomonas is a major asset, which will allow us to make rapid progress on the understanding of the mechanisms governing PCD in Chlamydomonas and particularly those involving nitrosylation, light, ROS production, and metacaspases.

## Materials and methods

### Strains, media, and growth conditions

Chlamydomonas (*C. reinhardtii*) strain D66 (CC-4425) (Schnell and Lefebvre 1993) was used as a wild type for this study in addition to the wild-type strain of CMJ030 (CC-4533) from the CLiP library (Li et al. 2019), which was used as a reference for the mutants of *MCA-I* (Cre12.g517451; LMJ.RY0402.231745) and *MCA-II* (Cre03.g184700; LMJ.RY0402.135349). Cells were grown in liquid cultures mixotrophically in TAP medium (Gorman and Levine 1965) on a rotary shaker (120 rpm) under continuous light (40–60 *µ*mol photons/m^2^/s), at 25 °C. Stress treatments were done in 24-well plates in a 1 mL volume at a concentration of 4–8 × 10^6^ cells/mL. Rose bengal was purchased from Sigma-Aldrich, and GSNO was synthesized as described in Tagliani et al. (2021). Dead cells were detected using Evans blue (Sigma-Aldrich), as described in de Carpentier et al. (2022). The mean between the different biological replicates (from 6 to 8) was then calculated, along with the Sem and a Student's *t* test to determine whether the samples are statistically different.

### Plasmid construction and Chlamydomonas transformation

*MCA-II* and *MCA-I* coding sequences were domesticated for the position B2 of the plant MoClo syntax (Patron et al. 2015), synthesized (Twist Bioscience), and cloned into the universal level 0 acceptor plasmid pAGM9121 (Weber et al. 2011) leading to the pCM0-121 (MCA-II) and pCM0-123 (MCA-I) plasmids. The *Venus* (with RbcS2 intron 2) sequence was amplified in position B3 without any stop codon by PCR on pCM0-066 and cloned into pAGM9121 yielding pCM0-122. To obtain pCM1-030, which was used to express MCA-II-mVenus, the following were assembled into pICH47732: pCM0-010 (pPsaD + PsaD 5′ UTR), pCM0-121 (MCA-II), pCM0-122 (Venus), pCM0-087 (FMDV 2A) (Rasala et al. 2012), pCM0-092 (aph7″), and pCM0-114 (PsaD 3′ UTR + tPsaD) from the Chlamydomonas MoClo toolkit (Crozet et al. 2018) (Weber et al. 2011). To obtain pCM1-031, which was used to express MCA-I-mVenus, the following were assembled into pICH47732: pCM0-010 (pPsaD + PsaD 5′ UTR), pCM0-123 (MCA-I), pCM0-122 (Venus), pCM0-087 (FMDV 2A) (Rasala et al. 2012), pCM0-092 (aph7″), and pCM0-114 (PsaD 3′ UTR + tPsaD) from the Chlamydomonas MoClo toolkit (Crozet et al. 2018) (Weber et al. 2011). Chlamydomonas cells were then transformed by electroporation as described previously (Onishi and Pringle 2016) with minor modifications. A total of 500 ng of purified DNA resulting from the digestion of the plasmid with *Bbs*I or *Bsa*I were introduced into 240 *µ*L of cells concentrated at 2 × 10^8^ cells/mL, in CHES buffer (10 mM CHES pH 9.25, 40 mM sucrose, 10 mM sorbitol), at 16 °C for 20 min. The cells were then electroporated in a 0.4-cm cuvette at 600 V, 25 *µ*F, and no shunt resistance. After overnight recovery in TAP + 60 mM sucrose, the cells were plated on TAP medium containing agar (1.2% *w*/*v*) and hygromycin (pCM1-030 and pCM1-031) or blasticidin (pCMM-23) (de Carpentier et al. 2020). The clones obtained on agar medium are then transferred to a well containing 200 *µ*L of liquid TAP medium in 96-well plates, and the transformants with the highest mVenus fluorescence values were selected for further analysis.

### Fluorescence screening and microscopy

To find transformants expressing MCA-II-mVenus, MCA-I-mVenus, or the positive Venus control, 48 transformants from pCM1-030 (MCA-II-mVenus), pCM1-031 (MCA-I-mVenus), and pCMM-23 (de Carpentier et al. 2022) (Venus) were grown in a volume of 200 *µ*L in a 96-well plate and screened after 5 and 10 d for yellow fluorescent protein (YFP) fluorescence using the CLARIOstar Plus (BMG Labtech) plate reader. MCA-II-mVenus and MCA-I-mVenus transformants, showing the highest level of YFP fluorescence, were selected for further experiments. Fluorescence microscopy was performed with the Nikon Eclipse Ti microscope. The microscope is equipped with a SOLA Light Engine system (lumencolor), used at an intensity of 6%. mVenus fluorescence was detected at an excitation of 515 nm and emission between 528 and 558 nm.

NO and singlet oxygen were detected using the fluorescent probes DAF-FM (4-amino-5-methylamino-2′,7′-difluorofluorescein diacetate) and SOS, respectively, following the manufacturer’s instructions (Invitrogen).

To obtain highly accurate and reproducible fluorescence images and quantifications, we used the following macro: https://github.com/fdecarpentier/fluo_quantification.

### DNA ladder

Genomic DNA was extracted from 50 × 10^6^ cell following Dellaporta’s procedure (Dellaporta et al. 1983) then treated with RNase (NEB) for 15 min at 37 °C. To analyze the quality of the DNA, 10 *µ*g of DNA was run in a 2% Tris-borate-EDTA agarose gel.

### RT-PCR and RT-qPCR

RNA was extracted with the TRIzol reagent as previously described (de Carpentier et al. 2022), treated with RNase-free DNase (New England Biolabs), and reverse-transcribed following manufacturer's recommendations (the ProtoScript Taq RT-PCR kit, New England Biolabs). PCR was performed with the Quick-Load Taq kit (NEB) according to the manufacturer's recommendations. For RT-qPCR analysis, PCR was performed with the Luna universal master mix (NEB) according to the manufacturer's recommendations, using a CFX96 real-time system (Bio-Rad). Relative mRNA abundance was calculated using the comparative delta-Ct method (Livak and Schmittgen 2001) and normalized to the corresponding *RACK1* (Cre06.g278222) gene levels. The list of the primers used in this study is shown in Supplemental Table S2.

### Sample preparation for proteomic analyses

After 5 h treatment, the 6 mL cultures were centrifuged (5 min; 2,300 × *g*; 25 °C) and the supernatants discarded. Pelleted cells were washed with 1 mL of fresh TAP medium and centrifuged again under the same conditions. After removal of the supernatant, the pelleted cells were resuspended in 100 *µ*L of lysis buffer containing 50 mM Tris-HCl buffer pH 7.9, 1 mM EDTA, and a cocktail of protease inhibitors (SigmaFast; Sigma-Aldrich; Saint-Quentin Fallavier; France). Chlamydomonas cells were lysed in 1.5-mL microtubes in the presence of 100 *µ*L of glass beads (acid-washed; 425–600 *µ*m mesh; Sigma-Aldrich) by performing 3 cycles of bead beating (30 s; 6.5 m/s) with a FastPrep instrument (MP Biochemicals). Cell debris were removed by centrifugation (15 min; 15,000 rpm; 4 °C), and protein concentration of soluble extracts was determined by bicinchoninic acid assay (Sigma-Aldrich).

For the 4 biological samples of each condition, 37 *µ*L of soluble extract (50 *µ*g) were mixed with 11.1 mg of urea (6 M final concentration) to denature proteins. Then, protein disulfide bonds were reduced in the presence of 10 mM DTT for 30 min at 25 °C and protein cysteines were further alkylated by 30 mM iodoacetamide for 30 min at 25 °C in the dark. Iodoacetamide reagent in excess was quenched by adding 1 *µ*L of 200 mM DTT. Then, 1 *µ*g of Lys-C endoprotease (Promega) was added to each protein sample and protein digestion was performed in a thermomixer (Eppendorf) at 30 °C for 150 min in the dark. Further digestion was performed overnight at 30 °C in the dark after addition of 1 *µ*g of trypsin (Trypsin Gold, Promega). Proteases were inhibited by adding 1 *µ*L of formic acid, and peptides were desalted on C-18 reverse phase spin columns (Pierce Thermo Scientific) following the recommendations of the supplier. Peptides were eluted 2 times with 40 *µ*L of 0.1% (*v*/*v*) formic acid in 70% (*v*/*v*) CH_3_CN. Eluted fractions were pooled and concentrated to 15 *µ*L using a vacuum concentrator before further dilution with 100 *µ*L of buffer A (0.1% [*v*/*v*] formic acid in 3% [*v*/*v*] CH_3_CN).

### Mass spectrometry–based proteomic analyses

For the 2 conditions (i.e. ± 1 mM GSNO), 4 biological replicates were analyzed as technical triplicates on a Q Exactive Plus hybrid quadripole-orbitrap mass spectrometer (Thermo Fisher, San José, CA, USA) coupled to an Easy 1000 reverse phase nano-flow LC system (Proxeon) using the Easy nano-electrospray ion source (Thermo Fisher). Five microliters of peptide mixtures were injected onto an Acclaim PepMap precolumn (75 *µ*m × 2 cm, 3 *µ*m, 100 Å; Thermo Scientific) equilibrated in buffer A and separated at a constant flow rate of 250 nL/min on a PepMap RSLC C18 EASY-Spray column (75 *µ*m × 50 cm, 2 *µ*m, 100 Å; Thermo Scientific) with a 90 min gradient (0% to 20% B solvent (0.1% [*v*/*v*] formic acid in acetonitrile) in 70 min and 20% to 32% B solvent in 20 min). Data were acquired in Data Dependent mode as described in Pérez-Pérez et al. (2017).

### Proteomic data processing

Raw files were processed using MaxQuant and the Andromeda search engine (version 1.6.17.0; Tyanova et al. 2016) against the CC-4532 Chlamydomonas protein database (v6.1; 32,672 entries including 32,590 nuclear-, 74 chloroplast-, and 8 mitochondrial-encoded proteins) (Craig et al. 2023) and the contaminant database embedded in MaxQuant. Trypsin/P was chosen as cutting specificity and up to 2 missed cleavages per peptide were allowed. Mass tolerances were set to default values for an Orbitrap mass analyzer (i.e. 20 and 4.5 ppm for precursors for first and main searches and 20 ppm for fragments). Minimum number of peptides, razor + unique peptides, and unique peptides were set to 1, 2, and 1, respectively. Peptides were identified and quantified using the “match between run” default setting and a reverse database-based false discovery rate (FDR) below 1% for both peptides and proteins. The intensity of proteins was quantified and normalized according to the MaxLFQ method using unique and razor peptides (Tyanova et al. 2016). Statistical analysis was performed using the Perseus software (version 1.6.15.0). Reverse proteins, potential contaminants, and proteins identified only by site were filtered out. LFQ values were log2 transformed, and log2 LFQ intensity for each biological replicate was determined as the median of the log2 LFQ intensity of the corresponding technical replicates. Proteins quantified in <3 biological replicates of 1 condition were discarded. A Benjamini–Hochberg-corrected 2-sample Student's *t* test was performed for proteins displaying at least 3 valid log2 LFQ intensities in both conditions. Proteins with an FDR < 1% were considered as statistically differentially expressed, and for those proteins, the fold changes between the 4 biological replicates of the 2 conditions were calculated as the ratio of the LFQ intensity medians. For proteins having 4 quantitative values in 1 condition but no value in the other one, a Benjamini–Hochberg-corrected 2-sample Student's *t* test (FDR < 1%) was performed using MS/MS counts. Proteins with an FDR < 1% were considered as statistically differentially expressed.

### Accession numbers

Sequence data from this article can be found in the Phytozome database under accession numbers: MCA-I: Cre12.g517451 and MCA-II: Cre03.g184700.

## Supplementary Material

kiad618_Supplementary_Data

## Supplemental data

The following materials are available in the online version of this article.

**Supplemental Figure S1**. Characteristics of strains overexpressing *MCA-I-mVenus* and *MCA-II-mVenus*.

**Supplemental Figure S2**. Analysis of *MCA-II-mVenus* overexpression according to culture conditions.

**Supplemental Table S1**. List of proteins deregulated at least twice during GSNO-induced PCD.

**Supplemental Table S2**. List of primers used in this study.

## Dive Curated Terms

The following phenotypic, genotypic, and functional terms are of significance to the work described in this paper:

CHLI2 Gramene: Cre12.g510800
CHLI2 Araport: Cre12.g510800

CHLI1 Gramene: Cre06.g306300
CHLI1 Araport: Cre06.g306300

CHLD Gramene: Cre05.g242000
CHLD Araport: Cre05.g242000

POR1 Gramene: Cre01.g015350
POR1 Araport: Cre01.g015350

CHLH1 Gramene: Cre07.g325500
CHLH1 Araport: Cre07.g325500
