# Supplementary material for: Type II metacaspase mediates light-dependent programmed cell death in Chlamydomonas reinhardtii
Source: Plant Physiol. 2023 Nov 16;194(4):2648–62. doi: 10.1093/plphys/kiad618 (PMC10980519; doi:10.1093/plphys/kiad618)
Supplement: kiad618_Supplementary_Data [file kiad618_supplementary_data.zip › Supplemental Figures.pdf]

Supplemental Figure S1

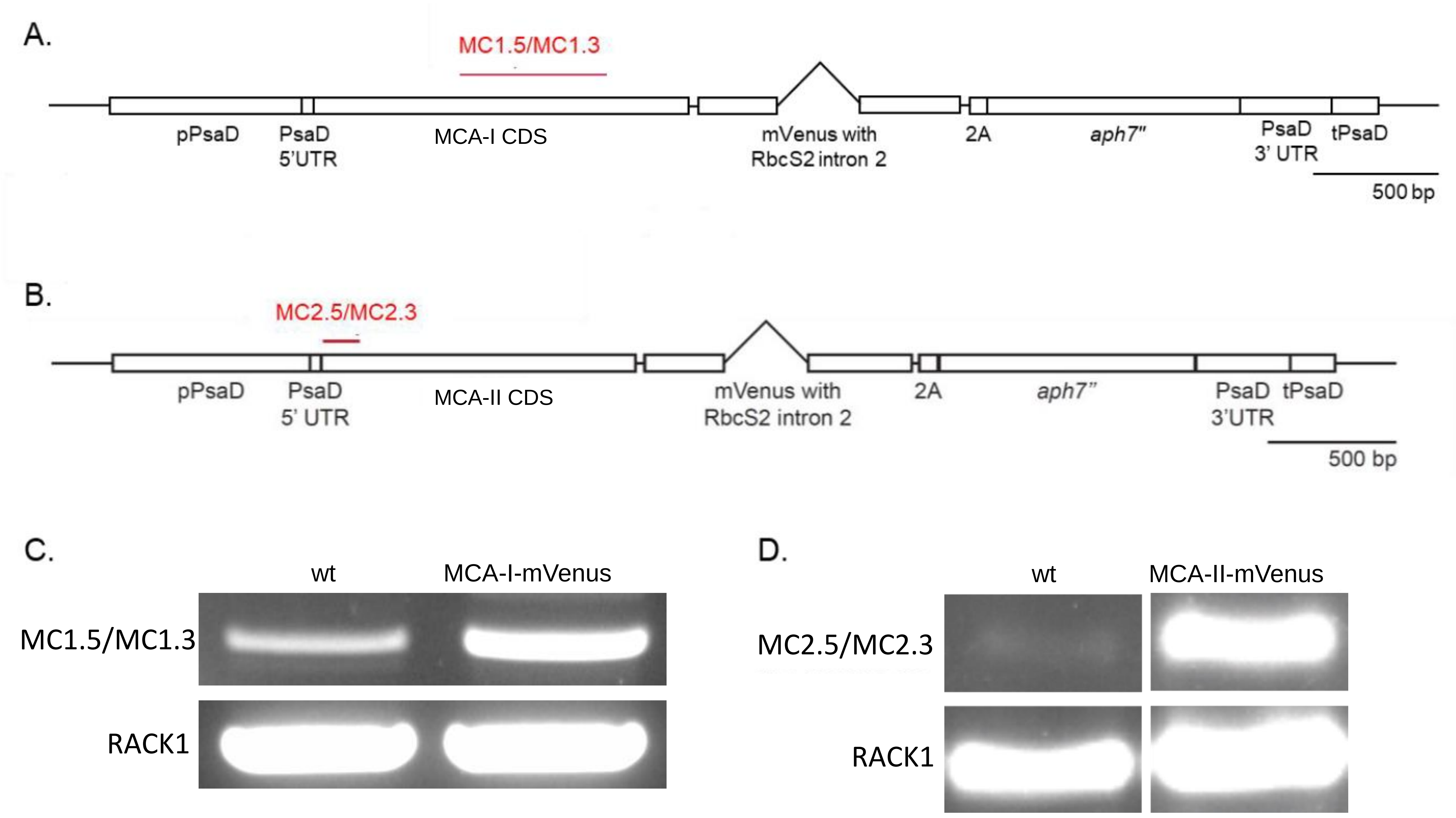

Supplemental Figure S1 : Characteristics of strains overexpressing MCA-I-mVenus and MCA-II-mVenus.
A, B Constructs used to express MCA-I-mVenus and MCA-II-mVenus, using the 2A system, which allows to express the gene of interest and the selection gene simultaneously. C, D Overexpression of MCA-I and MCA-II genes revealed by RT-PCR in MCA-I-mVenus and MCA-II-mVenus lines. Rack1 expression is used as an internal control.

Supplemental Figure S2

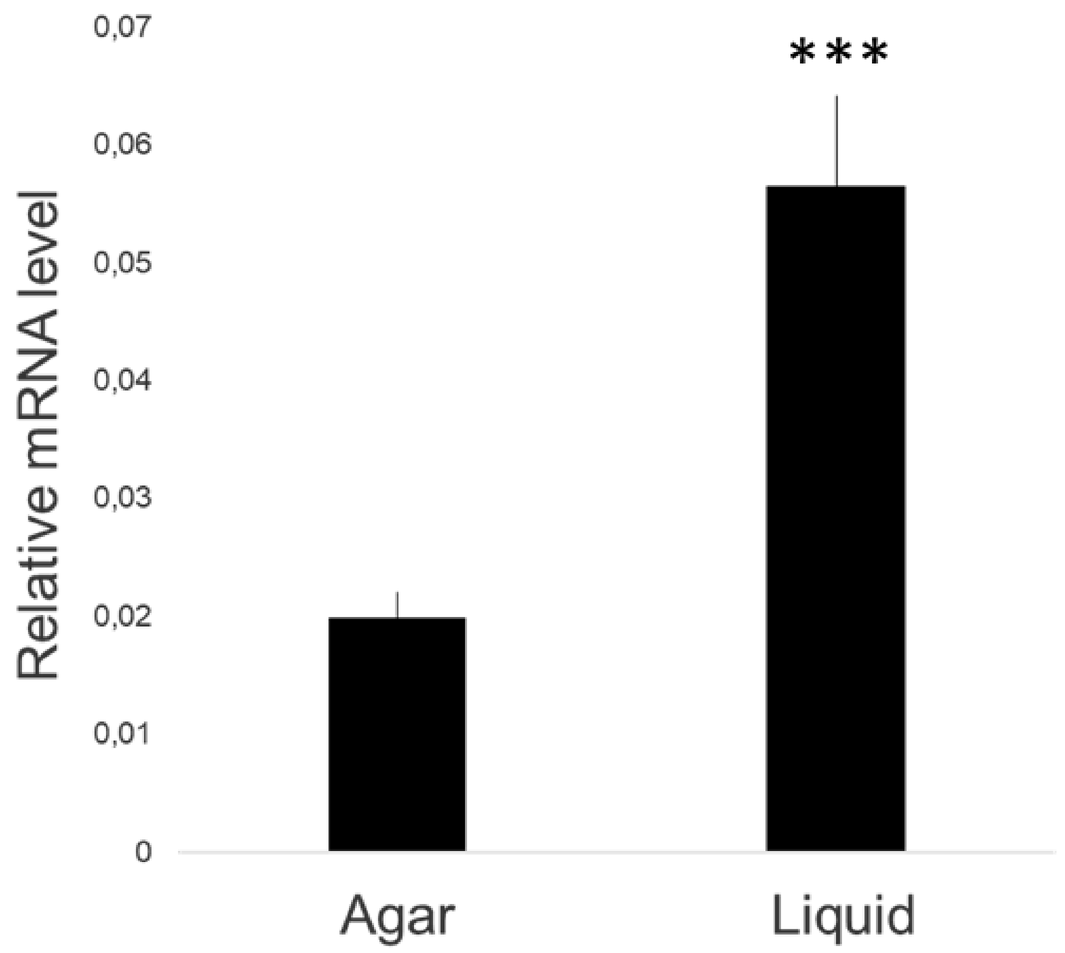

Supplemental Figure S2 : Analysis of MCA-II-mVenus overexpression according to culture conditions.

RNA from the MCA-II-mVenus strain was collected from cells grown on TAP agar medium (agar) or three days after transfer to TAP liquid medium (liquid). MCA-II expression was analyzed by quantitative RT-PCR, using MC2.5/MC2.3 primers. Values represent the average of 4 biological samples, error bars indicate ± SEM, and for t-test: *** $p \leq 0.001$.
